# SOCIAL MEDIA RESOURCES FOR BILINGUAL CAREGIVERS OF STROKE SURVIVORS

**DOI:** 10.1093/geroni/igz038.3252

**Published:** 2019-11-08

**Authors:** Sandra Sanchez-Reilly, Laura M Reilly-Sanchez, Valeria Restrepo, Marcos I Restrepo, Jeanette Ross, Michael J Mader

**Affiliations:** 1 South Texas Veterans Health Care System, San Antonio, Texas, United States; 2 Health Careers High School, San Antonio, Texas, United States; 3 Antonian High School, San Antonio, United States; 4 University of Texas Health Science Center San Antonio, San Antonio, Texas, United States

## Abstract

Background: Stroke survivors experience long-term disability also affecting informal caregivers (ICG). With current technology, social media might be the only way for ICG to gain training/access support. What resources are available for ICG of older adults who survived stroke (OASS)? Objective: To identify/analyze types of bilingual social media resources available to ICG of OASS Methods: Facebook data was bilingually collected (Spanish), including most popular groups and pages based on search engines containing terms such as stroke, CVA, caregiver. Similar numbers of groups (35 English vs. 52 Spanish) and pages (32 English vs. 34 Spanish) were analyzed. Data included pages and groups’ information, numbers-of-likes, type-of-organization and resources provided. Results: English-Facebook resources were more popular for pages and groups (3820/2010 vs. 190/7; p<0.001), Spanish resources were present, but with little activity among ICG. Majority of Spanish posts came from experts and English posts related to offering services or raising community awareness. Among both languages, pages provided resources related to social support (81%), improving caregiver skills (35%), advocacy (100%-English vs.56%-Spanish, p<0.001) and research news (84%-English vs.41%-Spanish, p<0.001). For English-ICG, more opportunities for live chats, messaging and inspirational messages were found (22-44% vs.3-9%, p<0.001). Conclusions: ICG of OASS could access Facebook resources to support multiple areas of caregiving including retrieving social support, gaining skills, learning new stroke-science findings and encountering live chats while getting inspired. Some resources are more available to English-ICG. Stroke-supporting organizations must consider using social media as crucial platforms to access bilingual resources and improve quality-of-life for ICG and OASS.

